# Bifunctional M13 Phage as Enzyme Container for the Reinforced Colorimetric–Photothermal Dual-Modal Sensing of Ochratoxin A

**DOI:** 10.3390/toxins15010005

**Published:** 2022-12-20

**Authors:** Weipeng Tong, Hanpeng Xiong, Hao Fang, Yuhao Wu, Haichuan Li, Xiaolin Huang, Yuankui Leng, Yonghua Xiong

**Affiliations:** 1 State Key Laboratory of Food Science and Technology, Nanchang University, Nanchang 330047, China; 2 School of Food Science and Technology, Nanchang University, Nanchang 330047, China; 3 Jiangxi-OAI Joint Research Institute, Nanchang University, Nanchang 330047, China

**Keywords:** bifunctional M13 bacteriophage, enzyme container, AuNP aggregation, ochratoxin A, plasmonic and photothermal ELISA

## Abstract

“Point of care” (POC) methods without expensive instruments and special technicians are greatly needed for high-throughput analysis of mycotoxins. In comparison, the most widely used screening method of the conventional enzyme-linked immunosorbent assay (ELISA) confronts low sensitivity and harmful competing antigens. Herein, we develop a plasmonic-photothermal ELISA that allows precise readout by color-temperature dual-modal signals based on enzymatic reaction-induced AuNP aggregation for highly sensitive detection of ochratoxin A (OTA). The bifunctional M13 phage carrying OTA that mimics the mimotope on the end of p3 proteins and abundant biotin molecules on the major p8 proteins is adopted as an eco-friendly competing antigen and enzyme container for amplifying the signal intensity. Under optimal conditions, both colorimetric and photothermal signals enable good dynamic linearity for quantitative OTA detection with the limits of detection at 12.1 and 8.6 pg mL^−1^, respectively. Additionally, the proposed ELISA was adapted to visual determination with a cutoff limit of 78 pg mL^−1^ according to a vivid color change from deep blue to red. The recoveries of OTA-spiked corn samples indicate the high accuracy and robustness of the proposed method. In conclusion, our proposed strategy provides a promising method for eco-friendly and sensitive POC screening of OTA. Moreover, it can be easily applied to other analytes by changing the involved specific mimotope sequence.

## 1. Introduction

Mycotoxin contamination in agricultural and food products is one of the major food safety concerns worldwide [1,2]. *Penicillium*- and *Aspergillus*-derived ochratoxin A (OTA) [3] is one of the most toxic mycotoxins that widely contaminate various crops (corn, wheat, beans, and cocoa), resulting in a serious threat to human health and consequential economic losses worldwide [4]. Many countries and international organizations have set strict limit standards for OTA residues [5,6], which are 0.5–10 µg kg^−1^ in different food commodities according to European Union regulations [7]. Regarding the universality and severe consequence of mycotoxin pollution, cheap “point of care” (POC) methods without expensive instruments and special technicians play a crucial role in the high-throughput analysis of mycotoxins [8]. Enzyme-linked immunosorbent assay (ELISA) is the most widely used POC method because of its merits of rapidity, simplicity, cost-effectiveness, and excellent specificity [9]. However, the conventional competitive ELISA for mycotoxin detection suffers from two major shortcomings [10,11]. First, the low-intensity and single-color colorimetric signal of enzymatic reaction-derived products leads to limited sensitivity and poor adaptability of on-site detection based on naked-eye interpretation in resource-constrained areas [12,13]. Second, using toxic target mycotoxin and organic reagents for preparing enzyme- or carrier protein-analyte conjugates as competing antigens leads to serious secondary pollution and a nonnegligible occupational hazard [14]. Hence, highly sensitive and eco-friendly immunoassays for mycotoxin screening must be developed urgently.

In recent decades, various colloidal nanoparticles have been used in developing biosensors to enhance or supersede current analytical techniques, making a great impact in research and practice applications [15,16]. In particular, plasmonic nanoparticles are interesting because their unique localized surface plasmon resonance (LSPR) enables them to produce intense responses to incident light [17], which can be linked to the presence of a target analyte to yield extremely sensitive detection [18,19]. Gold nanoparticles (AuNPs) are the most widely used plasmonic nanoparticles due to their merits, including easy preparation, excellent colloidal stability, and high accessibility for functionalization [20,21]. Highly sensitive AuNP-based sensing methods adopting various output signals, such as colorimetric signals [12], surface-enhanced Raman scattering [22], fluorescence [23], and photothermal signals [24] have been developed. AuNP-based colorimetric and photothermal sensing methods are particularly well suited for POC applications because of their simple signal readout via the naked eye or a thermometer [25]. Both colorimetric and photothermal signals generated from AuNPs are far more sensitive than those from conventional ELISAs because of the strong LSPR absorbance and photothermal effect of AuNPs. In recent years, plasmonic and photothermal ELISAs, which integrate LSPR modulation of AuNPs with conventional ELISA platforms, have attracted considerable attention because of their enhanced sensitivity [26]. The distinct color changes of plasmonic ELISAs are convenient for naked-eye readout without an excitation; the photothermal signal is more suitable than the colorimetric signal for muddy or colored samples [24]. Therefore, the combination of colorimetric and photothermal signals provides enhanced feasibility in POC applications and enhanced accuracy and reliability because of the mutual verification of the two signals [27]. Given that the two signals synchronously follow the LSPR modulation, dual modal plasmonic-photothermal ELISAs (ppELISAs) have been developed using AuNPs as signal transducers for the detection of disease biomarkers [28], nucleic acids [24], microorganisms [29], and mycotoxins [30]. A slight change in the compositions, shapes, sizes, or aggregation states of AuNPs may give rise to a remarkable LSPR variation. Among these modulation strategies, the AuNP aggregation-induced redshift of the LSPR absorbance always results in remarkably contrasting color changes and a distinct photothermal effect with a certain excitation. Previously, we reported a tyramine-mediated AuNP aggregation system based on the horseradish peroxidase (HRP)–catalyzed polymerization of phenolic hydroxyl in tyramine [31]. On this basis, a direct competitive ppELISA (dc-ppELISA) with enhanced sensitivity, accuracy, and reliability over conventional ELISAs can be promoted for mycotoxin detection.

The use of competing antigens of the enzyme-analyte conjugates in conventional direct competitive ELISAs for mycotoxin detection limits the sensitivity because one target molecule can only competitively inhibit the binding of one enzyme molecule. This approach also poses severe secondary pollution problems because of the consumption of toxic target mycotoxin and organic reagents. Therefore, novel competing antigens should be explored, particularly those that are eco-friendly and loaded with enhanced amounts of enzymes. Therefore, the M13 bacteriophage (M13 phage) carrying a mimic antigen epitope and multiple enzymes was proposed as a promising surrogate for competing antigens. The filamentous M13 is noninfectious to humans and composed of 2700 copies of the major p8 proteins and 3–5 copies of minor p3, p6, p7, and p9 proteins at the two ends [32]. It can be easily modified to mimic various target analytes, such as small molecules or proteins, by integrating mimotopes at the N-terminal of p3 proteins through a gene modification–fusion expression process [33]. The abundant copies of p8 proteins can be extensively functionalized as a container for high-density loading of signal transducers or regulators (e.g., fluorescent dyes and enzymes), resulting in remarkably enhanced signal intensities of biosensors [34]. Previously, we adopted an OTA-mimicking M13 phage (M13_OTA_) integrated with an OTA mimotope at the p3 proteins as an enzyme container to improve the sensitivity of a fluorescent ELISA for OTA detection [35].

Herein, we developed an eco-friendly dc-ppELISA for the highly sensitive detection of OTA in corn, and a colorimetric–photothermal dual model gave the developed method more applicability in POC screening. A biotinylated M13 phage (bio-M13_OTA_) was used as a competing antigen and container for glucose oxidase (GOx) enzymes, and citrate-AuNPs were applied as dual-modal signal transducers. The aggregation of citrate-capped AuNPs was induced by the HRP-catalyzed polymerization of tyramine, which was electrostatically adsorbed onto AuNPs in the presence of H_2_O_2_ produced from GOx-catalyzed oxidation of glucose. The analytical performance of the proposed dc-ppELISA, including the limit of detection (LOD), 50% competitive inhibition concentration (IC_50_), accuracy, and reliability, was evaluated and compared with that of conventional HRP-based ELISAs.

## 2. Results and Discussion

### 2.1. Principle of The Proposed dc-ppELISA Method

**Scheme 1:**
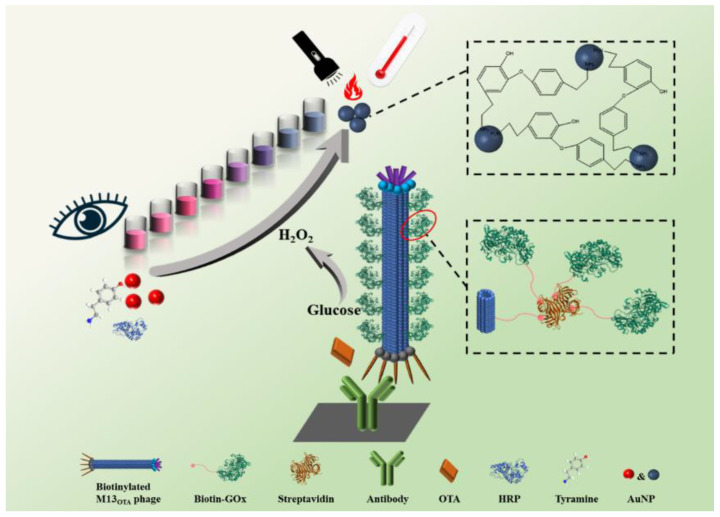
The principle of dc-ppELISA using a bifunctional M13 phage as a competing antigen for colorimetric–photothermal response arising from enzymatic reaction-induced AuNP aggregation.

In this study, we developed a novel dc-ppELISA for highly sensitive detection of OTA using a bio-M13_OTA_ phage as an eco-friendly competing antigen and enzyme container. Colorimetric–photothermal responses from enzymatic reaction-induced AuNP aggregation were also used as dual output signals (Scheme 1). In detail, the p8 proteins of M13_OTA_ are functionalized with abundant biotin molecules to enable the high-capacity loading of GOx enzymes via the biotin-streptavidin system. The bio-M13_OTA_ phage is captured by anti-OTA antibodies immobilized on the microplate in the absence of OTA. Then, large amounts of GOx are captured, generating a large amount of H_2_O_2_ via catalyzed oxidation of glucose. Afterward, H_2_O_2_ is catalyzed by HRP to produce hydroxyl radicals that can stimulate cross-linking among the phenolic hydroxyl moieties of tyramine. The tyramine is absorbed on the AuNP surface via the electrostatic interaction between the amino group of tyramine and the negative charge on citrate-capped AuNPs, thereby bringing AuNPs together. In OTA-positive samples, the binding of M13_OTA_ and AuNP aggregation is inhibited via a competitive binding process, resulting in a minor degree of AuNP aggregation.

### 2.2. Optimization of a Signal Transduction System Based on AuNP Aggregation

**Figure 1:**
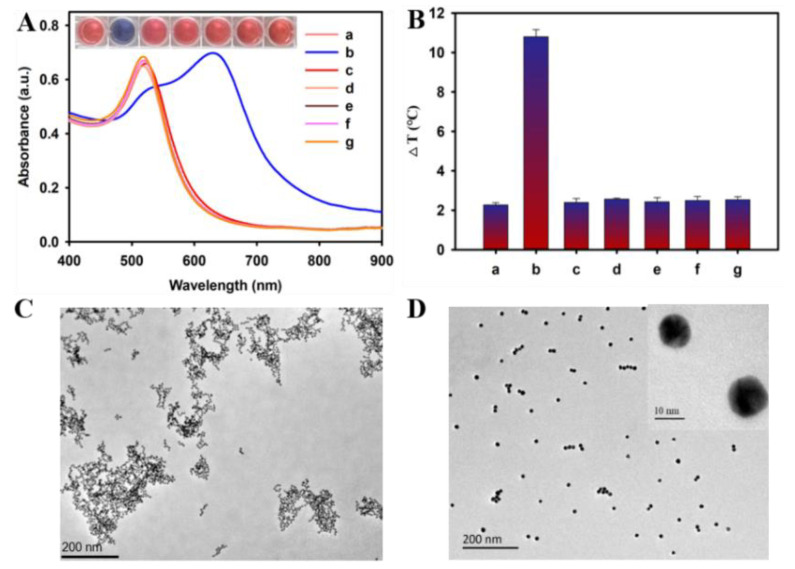
Characterization of colorimetric–photothermal dual signal response to AuNP aggregation induced by the HRP-H_2_O_2_-tyramine system. (**A**) LSPR spectra of different tests (a: H_2_O_2_ + TYR + AuNPs, b: H_2_O_2_ + HRP + TYR + AuNPs, c: HRP + TYR + AuNPs, d: HRP + H_2_O_2_ + AuNPs, e: H_2_O_2_ + AuNPs, f: TYR + AuNPs, g: HRP + AuNPs); (**B**) Temperature rise after 5 min excitation with 808 nm laser of test a, b, c, d, e, f, and g, and (**C**,**D**) TEM images of AuNP aggregation induced by the HRP-H_2_O_2_-tyramine system and dispersion of AuNPs in the absence of H_2_O_2_.

We first demonstrated the colorimetric–photothermal dual signal response to AuNP aggregation induced by the HRP-H_2_O_2_-tyramine system. Seven tests (a, b, c, d, e, f, and g) were conducted by adding one, two, or three components of the HRP-H_2_O_2_-tyramine system into a solution of 13 nm AuNPs. As shown in Figure 1A, only the addition of all HRP, H_2_O_2_, and tyramine (test b) results in a color change of the AuNP solution from red to blue, a redshift of LSPR from 520 to 630 nm, and a remarkable increase in LSPR absorbance in the range of 600–900 nm. Furthermore, the temperature of the reaction solution increases considerably after 5 min excitation with an 808 nm laser (Figure 1B). Conversely, the absence of any HRP-H_2_O_2_-tyramine system components provides no obvious signal change in the AuNP solution. The resultant AuNP solutions of test b and test g were further characterized. Figure 1C,D and Figure S1 show that in the presence of HRP and tyramine, the dispersive AuNPs in the solution form bulk agglomerations accompanied by a dramatically increased hydrodynamic particle size from 13.9 to 108.7 nm when H_2_O_2_ is added. All these results demonstrate the feasibility of dual-modal signal output arising from the AuNP aggregation induced by the HRP-H_2_O_2_-tyramine system. Moreover, H_2_O_2_ can be used as an effective signal regulator in the presence of HRP and tyramine.

The substrate solution of HRP-tyramine-AuNPs should be optimized to provide signal response toward low-concentration H_2_O_2_ and achieve a high sensing performance using the signal transduction system. In theory, a low tyramine concentration is conducive to achieving high sensitivity, whereas a high tyramine dosage can broaden the dynamic linearity of ppELISA. Nevertheless, tyramine overdosage may cause the self-aggregation of AuNPs due to charge neutralization. Therefore, the effect of tyramine dosage on AuNP aggregation was evaluated by adding different tyramine concentrations to the substrate solution containing 1.7 μg mL^−1^ of HRP and 5.3 nM of AuNPs. The ratio of the OD values at 630 and 520 nm (OD_630nm_/OD_520nm_) was chosen as the quantitative index for the AuNP aggregation. As shown in Figure S2A, the color of the AuNP solution is red when the tyramine concentration in the substrate solution is less than 33.3 µg mL^−1^. Conversely, when the tyramine concentration increases from 33.3 µg mL^−1^ to 267 µg mL^−1^, the color of the AuNP solution obviously changes from red to blue, and the OD_630nm_/OD_520nm_ value increases sharply from 0.2 to 1.1. These results show that an excess of tyramine can induce AuNP self-aggregation in the absence of H_2_O_2_. Moreover, 33.3 µg mL^−1^ of tyramine dosage is optimal in the substrate solution. Sufficient HRP is also vital in the signal transduction system. However, excess HRP may be absorbed on the AuNP surface, thereby deteriorating the AuNP aggregation triggered by the HRP-H_2_O_2_-tyramine system. The HRP dosage in the substrate solution was optimized by incubating different HRP concentrations in the substrate solution containing 33.3 µg mL^−1^ tyramine, 5.3 nM AuNPs, and 4 μM H_2_O_2_. The results in Figure S2B show that OD_630nm_/OD_520nm_ increases remarkably when the HRP final concentration increases from 0 to 1.67 µg mL^−1^, and the color of the AuNP solution varies from red to deep blue. Nevertheless, the OD_630nm_/OD_520nm_ value decreases gradually when the HRP concentration further increases from 1.7 µg mL^−1^ to 26.7 µg mL^−1^. Thus, 1.7 µg mL^−1^ of HPR in the substrate solution was used for all the succeeding studies.

**Figure 2:**
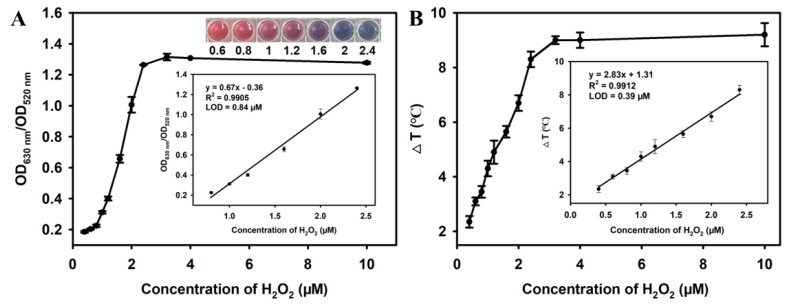
Dual signal response of the substrate solution to the addition of H_2_O_2_. (**A**) Calibration curve plotting OD_630 nm_/OD_520 nm_ against the concentration of H_2_O_2_; (**B**) Calibration curve plotting temperature variation against the concentration of H_2_O_2_.

Additionally, the sensitivity of the signal transduction system to H_2_O_2_ is a key factor in determining the H_2_O_2_-dependent immunoassay sensitivity. Thus, the responses of colorimetric and photothermal signal outputs to H_2_O_2_ were assessed in the optimal AuNP, tyramine, and HPR concentrations. As shown in Figure 2A, OD_630nm_/OD_520nm_ increases with the increase of H_2_O_2_ concentration from 0.4 to 3.2 μM. Then, it reaches a plateau with further increasing H_2_O_2_. A naked-eye cutoff limit of 1.6 μM for H_2_O_2_ is achieved, which is addressed by a distinct color change from red to purple at this point. The corresponding regression equation in the linear detection range of 0.8–2.4 μM can be expressed by y = 0.7x − 0.4 (R^2^ = 0.9905), and the LOD for H_2_O_2_ detection is calculated as 0.8 μM (Figure 2A). After 5 min exposure under 808 nm excitation, the redshift of LSPR and the increase in the absorbance at 808 nm arising from AuNP aggregation led to a distinct upregulated photothermal response (Figure 2B). The increase in temperature induced by the photothermal effect increases from 2.3 to 9.2 °C with the H_2_O_2_ increase from 0.4 to 4 μM. The corresponding regression equation in the linear detection range of 0.4–2.4 μM can be expressed by y = 2.8x + 1.3 (R^2^ = 0.9912), and the LOD for H_2_O_2_ detection is calculated as 0.4 μM. The above results imply that compared with the plasmonic signal, the photothermal signal can provide enhanced sensitivity for the immunoassay.

### 2.3. Development of The dc-ppELISA Method

**Figure 3:**
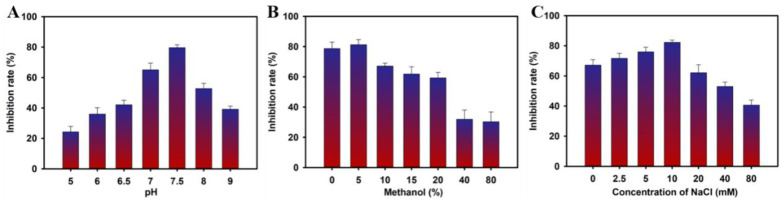
Optimization of the experimental conditions for the dc-ppELISA. (**A**) effect of pH values (6.5–8.0); (**B**) methanol content (*v*/*v*, 0–80%); (**C**) NaCl concentration (0~80 mM) on the performance of the dc-ppELISA. The error bars represent the standard deviation of the three measurements.

Thereafter, we developed a dc-ppELISA using a bifunctional phage (biotin-M13_OTA_) as a competing antigen, where the OTA-mimicking peptides were expressed at the end of p3 proteins. Moreover, numerous biotin molecules were modified on the major p8 proteins. According to our previous report, the as-prepared bio-M13_OTA_ exhibits a high loading capacity of 269 streptavidin molecules [35]. This finding indicates that biotin-M13_OTA_ can be used as the enzyme container for carrying a mass of biotinylated GOx (biotin-GOx). Unpurified anti-OTA ascitic fluids were used to coat the plate wells directly to maintain the maximum bio-activity of anti-OTA antibodies, and the photothermal mode have been used to proof the feasibility (Figure S3). Moreover, the concentrations of anti-OTA ascites and bifunctional phages were optimized via a checkerboard titration method. The competitive inhibition rate based on the colorimetric signal was used to evaluate the optimal parameters. As shown in Table S1, the proposed dc-ppELISA exhibits the maximum competitive inhibition rate at 82.4% for 1 ng mL^−1^ OTA when the concentrations of anti-OTA ascites and bio-M13_OTA_ are 0.8 μg mL^−1^ and 2 × 10^9^ pfu mL^−1^, respectively. Subsequently, the effects of pH, ionic strength, and methanol content of the sample solution were optimized to achieve the best detection performance of the dc-ppELISA. As shown in Figure 3A, the competitive inhibition rate of 1.0 ng mL^−1^ OTA in the PB buffer increases as pH increases from 5.0 to 7.5. It then decreases with a higher pH value. Methanol is a typically organic phase in the extraction solution for the hydrophobic mycotoxins. The high methanol concentration may interfere with immunoreaction efficiency. The results in Figure 3B show that the inhibition rate of PB (pH = 7.5) is unchanged when methanol content is less than 5%. However, it obviously decreases when the methanol content exceeds 5%. Hence, the highest methanol content in the sample solution should be less than 5%. The results in Figure 3C show that 10 mM NaCl in the sample solution (pH = 7.5, 5% methanol) provides the highest competitive inhibition rate. Therefore, the corn extract sample using 80% methanol solution (*v/v* in water) should be diluted with 10 mM PB buffer containing 10 mM NaCl (pH = 7.5) to methanol, final concentration of 5%.

### 2.4. Analytical Performance of The Proposed dc-ppELISA Method

**Figure 4:**
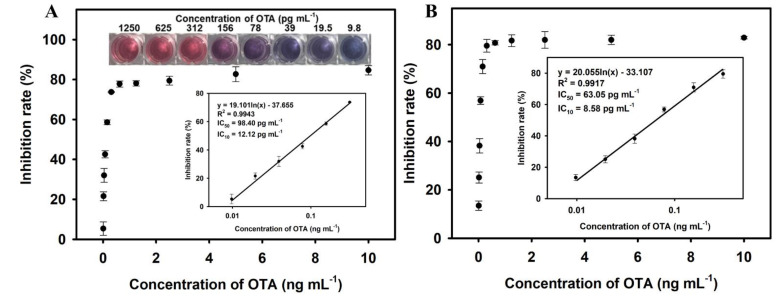
Competitive inhibition curves of the proposed dc-ppELISA based on colorimetric signal (**A**) and photothermal signal (**B**). The inset photos are the results of color development from detecting samples with different OTA concentrations.

Under optimal conditions, competitive inhibition curves based on the dual readout were obtained by detecting standard OTA samples and plotting the resultant competitive inhibition rates against OTA concentration. Figure 4A shows a sudden color change from blue to purple at an OTA concentration of 78 pg mL^−1^, which corresponds to 6.24 μg kg^−1^, regarded as the naked-eye cutoff limit. The corresponding regression equation in the linear range of colorimetric sensing from 9.8 pg mL^−1^ to 312 pg mL^−1^ can be expressed as y = 19.1 ln(x)–37.7 (R^2^ = 0.9943). The half-inhibition concentration (IC_50_) and 10% competitive inhibition concentration (IC_10_, equal to LOD) were calculated to be 98.4 pg mL^−1^ and 12.1 pg mL^−1^, which correspond to 7.9 μg kg^−1^ and 1 μg kg^−1^, respectively. Figure 4B shows the competitive inhibition curve based on the photothermal readout. The corresponding regression equation in a linear range from 9.8 pg mL^−1^ to 312 pg mL^−1^ can be expressed as y = 20.1 ln(x)–33.1 (R^2^ = 0.9917). The IC_50_ and IC_10_ based on the photothermal signal are calculated to be 63.1 pg mL^−1^ and 8.6 pg mL^−1^, which correspond to 5.0 μg kg^−1^ and 0.7 μg kg^−1^, respectively. The LOD values of the dc-ppELISA based on the colorimetric and photothermal signals are 12.3- and 17.5-fold lower than those of the conventional HRP-based ELISA (0.15 ng mL^−1^, Figure S4). This finding demonstrates the high potential of our proposed method for the sensitive detection of OTA in resource-limited regions.

Later, the specificity of the proposed assay was determined by detecting several other common mycotoxins, including AFB_1_, DON, ZEN, FB_1_, and CIT. The results in Figure S5 show the negligible signal response of the proposed dc-ppELISA to AFB_1_, DON, ZEN, FB_1_, and CIT with a high concentration at 1000 ng mL^−1^, indicating a cross-reactivity lower than 1‰. These results indicate the excellent selectivity of our proposed method for OTA detection.

The accuracy and precision of the dc-ppELISA method were determined by recovery evaluation from the OTA-fortified corn samples (2–100 μg kg^−1^). As shown in Table 1, the average recoveries (n = 3) based on the colorimetric readout in intra-assays and inter-assays range from 89.7% to 116.3% and from 95.4% to 119.4%, respectively; the average recoveries based on the temperature readout in intra-assays and inter-assays range from 95.4% to 113.6% and from 91.5% to 104.7%, respectively. These results indicate that our method can accurately detect OTA in real corn samples. The relative standard derivation for all the assays is less than 16%, revealing the good precision of our proposed method. Subsequently, 10 OTA-contaminated real corn samples were blindly analyzed through ultraperformance liquid chromatography-fluorescence detection (UPLC-FLD) method, and the results verified the practicability of our proposed method. Table 2 shows that all the detection results of our proposed method are basically consistent with those of the UPLC-FLD method, indicating that the proposed dc-ppELISA method is reliable for OTA screening in real corn samples.

## 3. Conclusions

In conclusion, a plasmonic and photothermal immunoassay for OTA screening was developed using a bio-M13_OTA_ phage as a green competing antigen. A dual readout signal transduction system based on the HRP-tyramine-H_2_O_2_ system gave the developed ELISA method more applicability in POC screening by replacing testing instruments. This novel method can achieve highly sensitive OTA detection based on a vivid color change that can be addressed by the naked eye, the redshift of LSPR spectra, and a temperature rise that can be read by a thermometer. The LODs based on colorimetric and photothermal signals are 12.3- and 17.5-fold lower than those of conventional HRP-based ELISAs. The results also demonstrate that our proposed method exhibits excellent selectivity, good accuracy, acceptable precision, and high reliability for sensing OTA in real corn samples. Although the detection time is increased compared with conventional ELISAs, we believe that the increase in time cost is acceptable compared with the improvement in sensing performance. We think that future work can be carried out from the efficient and rapid combination of enzymes and bacteriophages (such as click chemistry) to reduce the time cost. In summary, we proposed a promising POC screening strategy for the highly sensitive detection of OTA, and this strategy can be applied for other mycotoxin and contaminant screening by panning different mimotopes or ligands to the target analytes from phage display libraries.

## 4. Materials and Methods

### 4.1. Materials and Instruments

The following reagents were used: ochratoxin A (OTA), aflatoxin B_1_ (AFB_1_), fumonisin B_1_ (FB_1_), zearalenone (ZEN), deoxynivalenol (DON) and citrinin (CIT) (Huaan Magnech Bio-Tech., Beijing, China); HRP, GOx, bovine serum albumin (BSA), streptavidin, chloroauric Acid (HAuCl_4_) and trisodium citrate (Sigma-Aldrich, St. Louis, MO, USA); tyramine (Solarbio, Beijing, China); anti-OTA ascitic fluids (Wuxi Zodoboer Biotech., Wuxi, China); sulfosuccinimidyl 6-(biotinamido) hexanoate (Sulfo-NHS-LC-Biotin, NHS-biotin; Macklin, Shanghai, China); M13 bacteriophage with an OTA-mimicking peptide sequence of GMSWMMA (M13_OTA_) was given by Prof. Xuelan Chen of Jiangxi Normal University (Nanchang, China). All other analytical-grade chemicals were purchased from Sinopharm Chemical Corp. (Shanghai, China) and applied without further purification. We obtained 96-well plates from Corning, Inc. (New York, NY, USA); 18.2 MΩ cm deionized water was prepared using a Millipore Milli-Q water purification system (Millipore, Milford, MA, USA).

Transmission electron microscopy (TEM) images were obtained with a JEOL JEM-2100 electron microscope (Tokyo, Japan). Ultraviolet–visible (UV-Vis) absorption spectra were recorded using a double-beam UV-vis spectrophotometer (Cintra 10e; GBC, VI, Melbourne, Australia). Average hydrodynamic diameter was determined via dynamic light scattering (DLS) particle size analyzer (Zeta Sizer Nano ZS90, Malvern Instruments Ltd., Worcestershire, UK). The surface plasmon resonance (SPR) signal intensity of the AuNPs was determined using a Multiskan GO multimode reader (Thermo Fisher, Vantaa, Finland). Temperature of the AuNP solution under the excitation of an 808 nm laser was measured by an infrared camera, and all measurements were performed at room temperature.

### 4.2. Synthesis of Citrate-capped AuNPs

Citrate-capped AuNPs with an average diameter of 13 nm were synthesized via a one-step reduction method. Briefly, 250 mL of 1 mM HAuCl_4_ solution was heated to a boil under vigorous stirring, and 25 mL of 38.8 mM citrate solution was quickly added followed by 15 min of reaction under a stirring and boiling condition. During this period, the solution color changed immediately from pale yellow to blue and then gradually to dark red. Subsequently, the reaction solution was placed in an ice bath for 15 min, and the resultant AuNP solution was filtered with a 220 nm microporous membrane and stored at 4 °C for later use. The concentration of AuNPs was calculated to be 15.9 nM according to Beer’s Law.

### 4.3. Propagation of M13_OTA_ Bacteriophage

*Escherichia coli* ER2738 cells were cultured at 37 °C overnight in tetracycline-containing (10 μg mL^−^^1^) Luria–Bertani (LB) medium. Then, 200 μL of the above LB with enriched *Escherichia coli* cells and 1 μL of M13_OTA_ bacteriophage solution was inoculated into 20 mL of LB solution and cultured at 37 °C for 4~5 h under constant shaking at a speed of 250 rpm. Subsequently, the cell debris was removed via 10 min centrifugation at 5000 rpm, and then the proliferated M13_OTA_ in the supernatant was precipitated at 4 °C overnight after adding a PEG-NaCl solution (2.5 M NaCl, 20% PEG-8000). Thereafter, the precipitates of M13_OTA_ were collected via centrifugation (13,500 rpm, 10 min) and resuspended in 1 mL of phosphate-buffered (PB) solution. The cell debris was removed by spinning for 10 min at 5000 rpm. Then, 200 μL of PEG-NaCl solution was added to the supernatant for 1 h incubation on ice. The M13_OTA_ phage was obtained via centrifugation (13,500 rpm, 10 min) and resuspended with 200 μL of PB buffer. The concentration of amplified M13_OTA_ phage bacteriophages was determined via a plate count method.

### 4.4. Preparation of Bio-M13_OTA_ and Bio-GOx

Bio-M13_OTA_ and biotinylated GOx (bio-GOx) were obtained via the coupling between the amine groups on p8 proteins of the phage or GOx and the active ester group of NHS-biotin. During the preparation of bio-M13_OTA_ at the different molar ratios, different volumes of NHS-biotin (1 mg mL^−1^, dissolved in DMF) were added to 1 mL of M13_OTA_ solution (2 × 10^9^ pfu mL^−1^, pH = 8.6) After 4 h incubation on ice under vigorous stirring, 200 μL of PEG-NaCl solution was added for another 1 h incubation on ice. The bio-M13_OTA_ phage was purified via centrifugation (13,500 rpm, 10 min), resuspended in 1 mL of PB buffer, and stored at 4 °C. Similarly, for the preparation of bio-GOx at the molar ratio of 20:1, 74 μL of NHS-biotin (1 mg mL^−1^, dissolved in DMF) and 1 mg of GOx powder were mixed in 1 mL PBS solution (0.01 M, pH 8.6) and incubated on ice for 4 h under vigorous stirring. Excess NHS-biotin was removed via dialysis in PBS (0.01 M, pH 7.4) for 72 h. The resultant bio-GOx solution was stored at −20 °C with some glycerin added.

### 4.5. dc-ppELISA Procedure for OTA

The procedure for the dc-ppELISA using bio-M13_OTA_ as a competing antigen is as follows. First, 100 µL of protein G (25 µg mL^−1^, 0.01 M PBS pH 8.6) is added into each well of a 96-well microplate and incubated overnight at 4 °C. After washing three times with PBST (PBS containing 0.05% Tween 20) and once with PBS, 100 μL of anti-OTA ascitic fluids (0.75 μg mL^−1^ in PBS, pH = 8.6) is added to each well and incubated for 2 h at 37 °C. After removing excess anti-OTA ascitic fluids, 300 μL of blocking buffer (1% BSA in 0.01M PBS 7.4) is added for another 2 h incubation at 37 °C. After a repeated washing process, 50 μL of the sample solution and 50 μL of bio-M13_OTA_ solution (2 × 10^9^ pfu mL^−1^) are added to each well. After incubating for 60 min at 37 °C, the microplate is washed again. Then, 100 μL of streptavidin solution is added and incubated for 30 min at 37 °C. After washing away the unbound streptavidin, 100 μL of bio-GOx is added and incubated for another 30 min at 37 °C. After unreacted bio-GOx is wiped off, 100 μL of D (+)-glucose (1 mg mL^−1^ in PBS, pH = 7.4) is added for 1 h incubation at 37 °C. Finally, 150 μL of substrate solution containing HRP (50 μL, 5 µg mL^−1^), tyramine (50 μL, 100 µg mL^−1^), and AuNPs (50 μL, 15.9 nM) is added into each well. After 5 min incubation, the microplate is photographed to record the color development and the optical density (OD) of each well at 520 and 630 nm are detected by multimode microplate reader. Furthermore, an infrared camera is used to record the temperature of each well after irradiation with an 808 nm laser for another 5 min. The inhibition rates were calculated according to the following formula: inhibition rate (%) = (1 − B/B_0_) * 100%, where B and B_0_ represent OD_630nm_/OD_520nm_ values (colorimetric signal) or temperature changes (∆T, photothermal signal) obtained from detecting OTA-positive and -negative samples.

### 4.6. Sample Preparation

Corn samples were purchased from grain procurement agencies (Shandong, China), and verified to be OTA-free via high-performance liquid chromatography (HPLC). The HPLC method was according to the Chinese national standard GB 5009.96-2016, which is listed below: (a) chromatographic column: C18 column, column length 150 mm, inner diameter 4.6 mm, particle size 5 μm; (b) mobile phase: acetonitrile water glacial acetic acid (96 + 102 + 2); (c) flow rate: 1.0 mL/min; (d) column temperature: 35 °C; (e) injection volume: 50 μL; (f) detection wavelength: excitation wavelength 333 nm, emission wavelength 460 nm. All samples were thoroughly ground and mixed before use, and the corn samples for assay validation were prepared following a previously reported method and Chinese national standard GB 5009.96-2016. In brief, several portions of finely ground corn samples (5.0 g each portion) were spiked with OTA at different levels (2–100 μg kg^−1^). Each portion of OTA-spiked corn sample was extracted with 25 mL methanol-H_2_O solution (80% methanol: 20% H_2_O) under vigorous shaking for 20 min. After centrifugation at 10,000 rpm for 5 min, the supernatant was then collected and stored at 4 °C for further use.

## Figures and Tables

**Table 1:** Recoveries of OTA-spiked corn samples using our proposed dc-ppELISA.

Signal	OTA Added (μg kg^−1^)	Mean ± SD (μg kg^−1^)	Recovery (%)	CV (%)	Mean ± SD (μg kg^−1^)	Recovery (%)	CV (%)
		Intra-Assay (n = 3)			Inter-Assay (n = 3)		
plasmonic	2	2.3 ± 0.3	116.3	11.3	2.4 ± 0.4	119.4	15.7
	40	35.9 ± 5.5	89.7	15.3	38.2 ± 4.8	95.4	12.5
	100	98.4 ± 13.2	98.5	13.5	103.2 ± 7.0	103.2	6.8
photothermal	2	2.3 ± 0.3	113.6	11.8	1.8 ± 0.2	91.6	8.2
	40	38.2 ± 3.0	95.4	8.0	39.5 ± 2.4	98.7	6.0
	100	108.0 ± 5.8	108.0	5.3	104.7 ± 14.2	104.7	13.6

**Table 2:** Comparison of results obtained from blindly detecting real corn samples using the proposed dc-ppELISA and UPLC-FLD method.

Incurred Samples	Plasmonic Signal (n = 3)		Photothermal Signal (n = 3)		HPLC (μg kg^−1^)
	Mean ± SD (μg kg^−1^)	CV (%)	Mean ± SD (μg kg^−1^)	CV (%)	
1	43.6 ± 2.4	5.4	40.9 ± 2.2	5.3	50.0
2	98.5 ± 13.2	13.5	108.0 ± 5.8	5.3	103.5
3	35.9 ± 5.5	15.3	38.2 ± 3.1	8.0	36.4
4	165.4 ± 18.2	11.0	157.7 ± 21.4	13.6	160.5
5	66.8 ± 7.0	10.5	61.3 ± 5.0	8.2	60.7
6	4.2 ± 0.2	4.8	4.7 ± 0.5	10.8	5.2
7	26.4 ± 1.0	3.8	30.8 ± 1.9	6.3	24.1
8	8.8 ± 1.0	11.7	11.2 ± 1.2	10.8	18.9
9	23.3 ± 2.6	11.3	22.7 ± 2.7	11.8	26.3
10	85.2 ± 10.4	12.2	77.3 ± 8.8	11.3	87.5

## Data Availability

Data is contained within the article or supplementary material.

## Supplementary Materials

The following are available online at https://www.mdpi.com/article/10.3390/toxins15010005/s1, Figure S1: Particle size of AuNP aggregation induced by HRP-H_2_O_2_-tyramine system and dispersion of AuNPs in the absence of H_2_O_2_; Figure S2: Optimization of the substrate solution of HRP-tyramine-AuNP signal transduction; Figure S3: Feasibility of adopting the biotinylated M13_OTA_ phage as competing antigen in the proposed dc-ppELISA for OTA detection; Figure S4: Cross-reactivity of the proposed dc-ppELISA toward other common mycotoxins; Figure S5: Calibration curve of conventional HRP-based ELISA; Table S1: Optimization of the working conditions of coating antibody and biotinylated M13_OTA_ phage using checkerboard method.

## References

[1] Wan J., Chen B., Rao J. (2020). Occurrence and preventive strategies to control mycotoxins in cereal-based food. Compr. Rev. Food Sci. Food Saf..

[2] Luo S., Du H., Kebede H., Liu Y., Xing F. (2021). Contamination status of major mycotoxins in agricultural product and food stuff in Europe. Food Control.

[3] Van der Merwe K., Steyn P., Fourie L., Scott D.B., Theron J. (1965). Ochratoxin A, a toxic metabolite produced by *Aspergillus ochraceus* Wilh. Nature.

[4] Reddy K., Salleh B., Saad B., Abbas H., Abel C., Shier W. (2010). An overview of mycotoxin contamination in foods and its implications for human health. Toxin Rev..

[5] Duarte S., Lino C., Pena A. (2010). Mycotoxin food and feed regulation and the specific case of ochratoxin A: A review of the worldwide status. Food Addit. Contam..

[6] Li X., Ma W., Ma Z., Zhang Q., Li H. (2022). Recent progress in determination of ochratoxin a in foods by chromatographic and mass spectrometry methods. Crit. Rev. Food Sci. Nutr..

[7] The European Commission (2010). Commission Regulation (EU) No 105/2010 of 5 February 2010 amending Regulation (EC) No 1881/2006 setting maximum levels for certain contaminants in foodstuffs as regards ochratoxin A. Off. J. Eur. Union.

[8] Shen L., Hagen J.A., Papautsky I. (2012). Point-of-care colorimetric detection with a smartphone. Lab Chip.

[9] Pei K., Xiong Y., Xu B., Wu K., Li X., Jiang H., Xiong Y. (2018). Colorimetric ELISA for ochratoxin A detection based on the urease-induced metallization of gold nanoflowers. Sens. Actuators B Chem..

[10] Xiong Y., Leng Y., Li X., Huang X., Xiong Y. (2020). Emerging strategies to enhance the sensitivity of competitive ELISA for detection of chemical contaminants in food samples. TrAC Trends Anal. Chem..

[11] Huang X., Chen R., Xu H., Lai W., Xiong Y. (2016). Nanospherical brush as catalase container for enhancing the detection sensitivity of competitive plasmonic ELISA. Anal. Chem..

[12] He Y., Tian F., Zhou J., Zhao Q., Fu R., Jiao B. (2020). Colorimetric aptasensor for ochratoxin A detection based on enzyme-induced gold nanoparticle aggregation. J. Hazard. Mater..

[13] Liu X., Xu Y., He Q.-H., He Z.-Y., Xiong Z.-P. (2013). Application of mimotope peptides of fumonisin b1 in Peptide ELISA. J. Agric. Food Chem..

[14] Song Z., Feng L., Leng Y., Huang M., Fang H., Tong W., Chen X., Xiong Y. (2021). Dramatically Enhancing the Sensitivity of Immunoassay for Ochratoxin A Detection by Cascade-Amplifying Enzyme Loading. Toxins.

[15] Jiang C., Lan L., Yao Y., Zhao F., Ping J. (2018). Recent progress in application of nanomaterial-enabled biosensors for ochratoxin A detection. TrAC Trends Anal. Chem..

[16] Rai M., Jogee P.S., Ingle A.P. (2015). Emerging nanotechnology for detection of mycotoxins in food and feed. Int. J. Food Sci. Nutr..

[17] Khlebtsov N.G., Dykman L.A. (2010). Optical properties and biomedical applications of plasmonic nanoparticles. J. Quant. Spectrosc. Radiat. Transf..

[18] Liu J., He H., Xiao D., Yin S., Ji W., Jiang S., Luo D., Wang B., Liu Y. (2018). Recent advances of plasmonic nanoparticles and their applications. Materials.

[19] Fong K.E., Yung L.-Y.L. (2013). Localized surface plasmon resonance: A unique property of plasmonic nanoparticles for nucleic acid detection. Nanoscale.

[20] Daniel M.-C., Astruc D. (2004). Gold nanoparticles: Assembly, supramolecular chemistry, quantum-size-related properties, and applications toward biology, catalysis, and nanotechnology. Chem. Rev..

[21] Jain P.K., Lee K.S., El-Sayed I.H., El-Sayed M.A. (2006). Calculated absorption and scattering properties of gold nanoparticles of different size, shape, and composition: Applications in biological imaging and biomedicine. J. Phys. Chem. B.

[22] Meng D., Ma W., Wu X., Xu C., Kuang H. (2020). DNA-Driven Two-Layer Core-Satellite Gold Nanostructures for Ultrasensitive MicroRNA Detection in Living Cells. Small.

[23] Yang Y., Hou J., Huo D., Wang X., Li J., Xu G., Bian M., He Q., Hou C., Yang M. (2019). Green emitting carbon dots for sensitive fluorometric determination of cartap based on its aggregation effect on gold nanoparticles. Microchim. Acta.

[24] Zhou W., Hu K., Kwee S., Tang L., Wang Z., Xia J., Li X. (2020). Gold nanoparticle aggregation-induced quantitative photothermal biosensing using a thermometer: A simple and universal biosensing platform. Anal. Chem..

[25] Tao Y., Luo F., Guo L., Qiu B., Lin Z. (2020). Target-triggered aggregation of gold nanoparticles for photothermal quantitative detection of adenosine using a thermometer as readout. Anal. Chim. Acta.

[26] Ding L., Shao X., Wang M., Zhang H., Lu L. (2021). Dual-mode immunoassay for diethylstilbestrol based on peroxidase activity and photothermal effect of black phosphorus-gold nanoparticle nanohybrids. Anal. Chim. Acta.

[27] Liu Y., Pan M., Wang W., Jiang Q., Wang F., Pang D.-W., Liu X. (2018). Plasmonic and photothermal immunoassay via enzyme-triggered crystal growth on gold nanostars. Anal. Chem..

[28] Wei Z., Yu Y., Hu S., Yi X., Wang J. (2021). Bifunctional Diblock DNA-Mediated Synthesis of Nanoflower-Shaped Photothermal Nanozymes for a Highly Sensitive Colorimetric Assay of Cancer Cells. ACS Appl. Mater. Interfaces.

[29] Zheng L., Dong W., Zheng C., Shen Y., Zhou R., Wei Z., Chen Z., Lou Y. (2022). Rapid photothermal detection of foodborne pathogens based on the aggregation of MPBA-AuNPs induced by MPBA using a thermometer as a readout. Colloids Surf. B Biointerfaces.

[30] Zhang T., Lei L., Tian M., Ren J., Lu Z., Liu Y., Liu Y. (2021). Multifunctional Fe_3_O_4_@Au supraparticle as a promising thermal contrast for an ultrasensitive lateral flow immunoassay. Talanta.

[31] Liang Y., Huang X., Chen X., Zhang W., Ping G., Xiong Y. (2018). Plasmonic ELISA for naked-eye detection of ochratoxin A based on the tyramine-H_2_O_2_ amplification system. Sens. Actuators B Chem..

[32] Molek P., Bratkovič T. (2015). Bacteriophages as scaffolds for bipartite display: Designing Swiss army knives on a nanoscale. Bioconjug. Chem..

[33] Wang X.S., Chen P.H.C., Hampton J.T., Tharp J.M., Reed C.A., Das S.K., Wang D.S., Hayatshahi H.S., Shen Y., Liu J. (2019). A genetically encoded, phage-displayed cyclic-peptide library. Angew. Chem..

[34] Blaik R.A., Lan E., Huang Y., Dunn B. (2016). Gold-coated M13 bacteriophage as a template for glucose oxidase biofuel cells with direct electron transfer. ACS Nano.

[35] Tong W., Fang H., Xiong H., Wei D., Leng Y., Hu X., Huang X., Xiong Y. (2021). Eco-Friendly Fluorescent ELISA Based on Bifunctional Phage for Ultrasensitive Detection of Ochratoxin A in Corn. Foods.

