# Supplementary material for: Bifunctional M13 Phage as Enzyme Container for the Reinforced Colorimetric–Photothermal Dual-Modal Sensing of Ochratoxin A
Source: Toxins (Basel). 2022 Dec 20;15(1):5. doi: 10.3390/toxins15010005 (PMC9867381; doi:10.3390/toxins15010005)
Supplement: Supplementary file 1 [file toxins-15-00005-s001.zip › toxins-2076225-supplementary.pdf]

toxins

MDPI

Article

Bifunctional M13 Phage as Enzyme Container for The Reinforced Colorimetric–Photothermal Dual-Modal Sensing of Ochratoxin A

Hanpeng Xiong, Weipeng Tong, Hao Fang, Yuhao Wu, Haichuan Li, Xiaolin Huang, Yuankui Leng and Yonghua Xiong

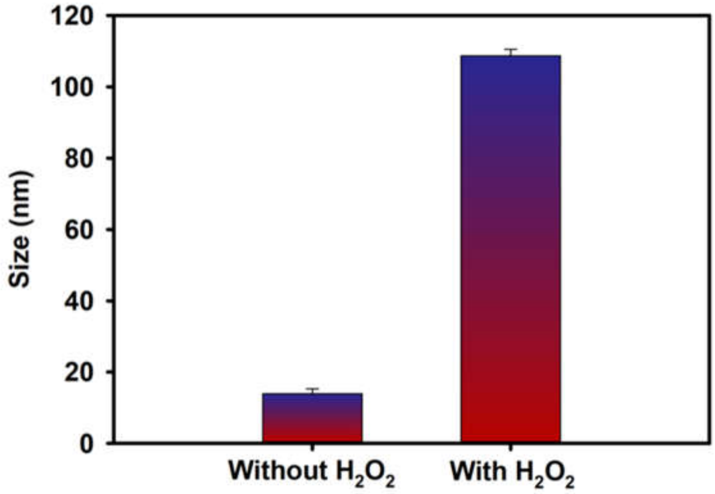

Figure S1. The particle size of AuNPs aggregation induced by HRP-H_2O_2-Tyramine system and dispersion of AuNPs in the absence of H_2O_2.

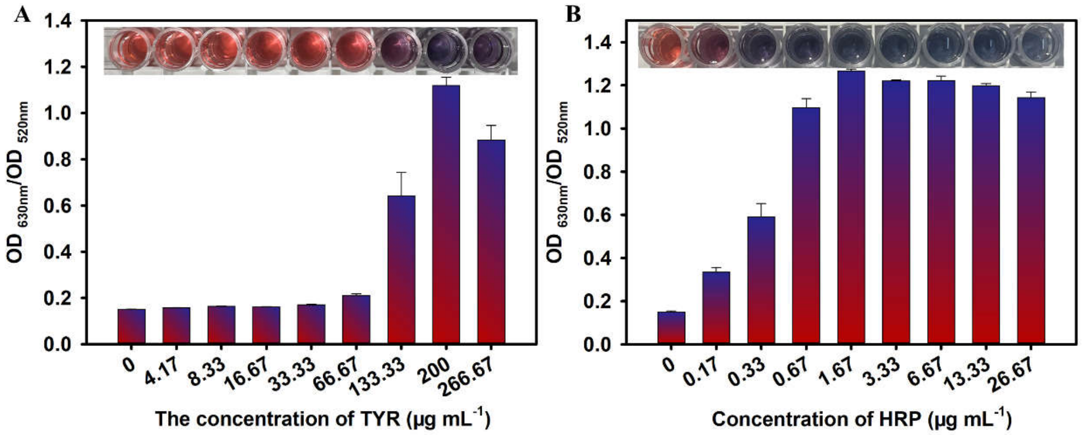

Figure S2. Optimization of the substrate solution of HRP-Tyramine-AuNPs signal transduction. (A) tyramine and (B) HRP.

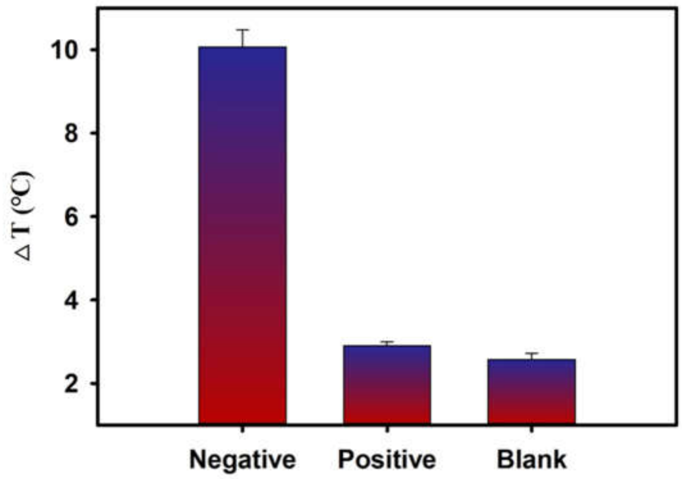

Figure S3. Feasibility to adopt the biotinylated M13$_{OTA}$ phage as competing antigen in the proposed dc-ppELISA for OTA detection. The blank group was conducted in the absence of biotinylated M13$_{OTA}$, the negative and positive groups were conducted with OTA negative and OTA positive (1 ng mL^{-1}) samples in the presence of biotinylated M13$_{OTA}$. The error bars represent the standard deviation of the three measurements.

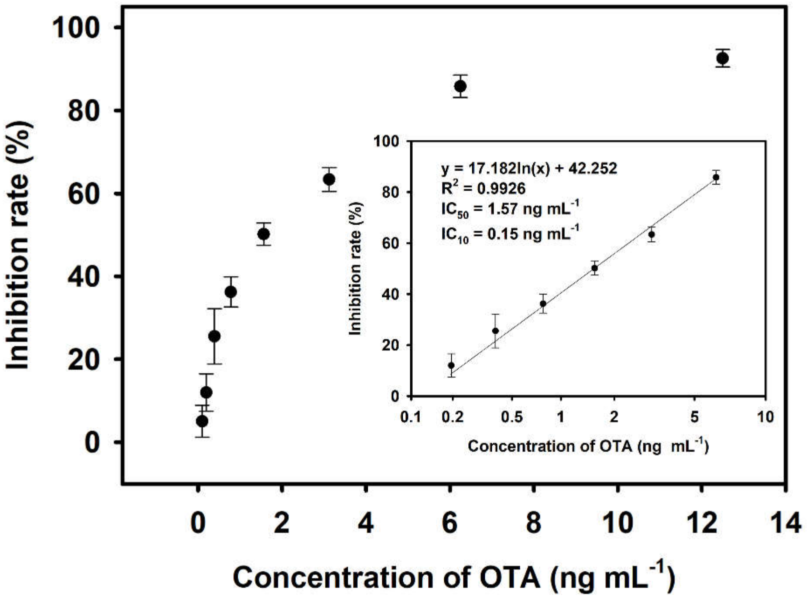

Figure S4. Calibration curve of conventional HRP-based ELISA, the inset shows a dynamic linear range of OTA concentrations from 0.098 ng mL^{-1} to 3.125 ng mL^{-1}. Each independent experiment was repeated 3 times.

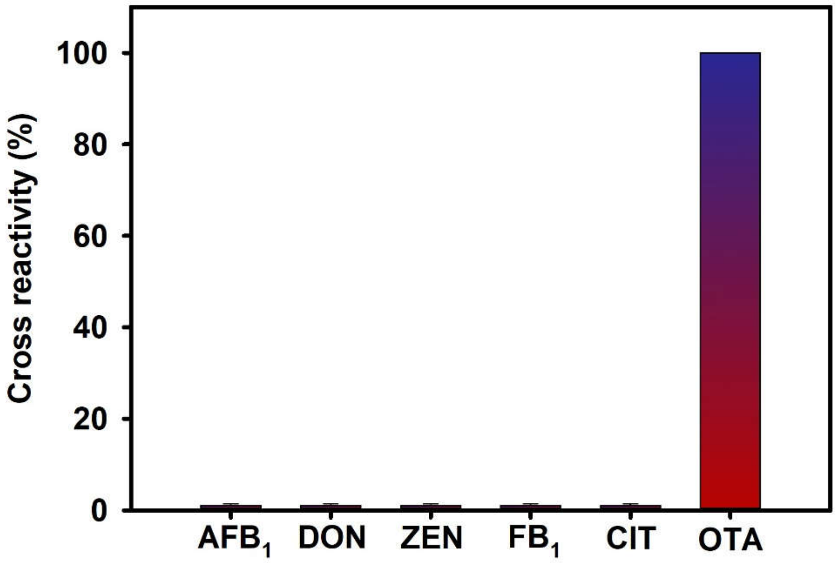

Figure S5. Cross-reactivity of the proposed dc-ppELISA toward other common mycotoxins. The samples detected are AFB_1, DON, ZEN, FB_1, CIT, and OTA. Error bars represent the standard deviation based on three measurements.

Table S1. Optimization of the working conditions of coating antibody and biotinylated $M13_{OTA}$ phage using checkerboard method.

Concentration of anti-OTA ascitic fluids (μg mL^{-1})	Concentration of Bio-phage (pfu mL^{-1})			
	4×10^9	2×10^9	1×10^9	5×10^8
3.00	52.52	54.07	65.48	70.66
1.50	79.17	75.00	65.74	66.59
0.75	75.20	82.41	76.54	38.58
0.38	75.60	67.43	45.05	68.02
